# Tetra­kis(2,6-diamino­pyridinium) diphthalate 2,6-diamino­pyridine

**DOI:** 10.1107/S1600536809044468

**Published:** 2009-10-31

**Authors:** Mohammad T. M. Al-Dajani, Abdusalam Salhin, Nornisah Mohamed, Wan-Sin Loh, Hoong-Kun Fun

**Affiliations:** a School of Pharmaceutical Sciences, Universiti Sains Malaysia, 11800 USM, Penang, Malaysia; b School of Chemical Sciences, Universiti Sains Malaysia, 11800 USM, Penang, Malaysia; c X-ray Crystallography Unit, School of Physics, Universiti Sains Malaysia, 11800 USM, Penang, Malaysia

## Abstract

In the title compound, 4C_5_H_8_N_3_^+^·2C_8_H_4_O_4_^2−^·C_5_H_7_N_3_, the asymmetric unit consists of two protonated diamino­pyridine cations, one phthalate anion and one half of a diamino­pyridine mol­ecule, which has twofold rotation symmetry and is disordered over two positions with a site-occupancy ratio of 0.534 (3):0.466 (3). In the disordered structure, both pyridine rings are essentially planar, with maximum deviations of 0.011 (2) and 0.006 (2) Å, and these two rings are inclined to one another at a dihedral angle of 79.86 (10)°. In the crystal structure, inter­molecular N—H⋯O and C—H⋯O hydrogen bonds link the ions and mol­ecules into a three-dimensional network. The structure is further stabilized by C—H⋯π inter­actions.

## Related literature

For background to 2,6-diamino­pyridines, see: Abu Zuhri & Cox (1989 ▶); Inuzuka & Fujimoto (1990 ▶). For background and the biological activity of phthalic acid, see: Brike *et al.* (2002 ▶); Yamamoto *et al.* (1990 ▶). For the preparation of polymer complexes, see: El-Mossalamy (2001 ▶). For a related structure: see: Büyükgüngör & Odabąsoğlu (2006 ▶). For bond-length data, see: Allen *et al.* (1987 ▶). For the stability of the temperature controller used for the data collection, see: Cosier & Glazer (1986 ▶).
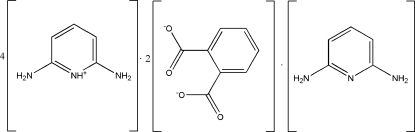


## Experimental

### 

#### Crystal data

4C_5_H_8_N_3_^+^·2C_8_H_4_O_4_^2−^·C_5_H_7_N_3_
*M* *_r_* = 877.94
Monoclinic, 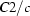

*a* = 29.7011 (6) Å
*b* = 15.2183 (3) Å
*c* = 9.7666 (2) Å
β = 101.670 (1)°
*V* = 4323.25 (15) Å^3^
*Z* = 4
Mo *K*α radiation
μ = 0.10 mm^−1^
*T* = 100 K
0.65 × 0.19 × 0.08 mm

#### Data collection

Bruker SMART APEXII CCD area-detector diffractometer
Absorption correction: multi-scan (**SADABS**; Bruker, 2005 ▶) *T*_min_ = 0.939, *T*_max_ = 0.992
28159 measured reflections
6403 independent reflections
3722 reflections with *I* > 2σ(*I*)
*R*_int_ = 0.037

#### Refinement

*R*[*F* ^2^ > 2σ(*F* ^2^)] = 0.064
*wR*(*F* ^2^) = 0.153
*S* = 1.05
6403 reflections
368 parameters
138 restraints
H atoms treated by a mixture of independent and constrained refinement
Δρ_max_ = 0.41 e Å^−3^
Δρ_min_ = −0.29 e Å^−3^

### 

Data collection: *APEX2* (Bruker, 2005 ▶); cell refinement: *SAINT* (Bruker, 2005 ▶); data reduction: *SAINT*; program(s) used to solve structure: *SHELXTL* (Sheldrick, 2008 ▶); program(s) used to refine structure: *SHELXTL*; molecular graphics: *SHELXTL*; software used to prepare material for publication: *SHELXTL* and *PLATON* (Spek, 2009 ▶).

## Supplementary Material

Crystal structure: contains datablocks global, I. DOI: 10.1107/S1600536809044468/is2475sup1.cif

Structure factors: contains datablocks I. DOI: 10.1107/S1600536809044468/is2475Isup2.hkl

Additional supplementary materials:  crystallographic information; 3D view; checkCIF report

## Figures and Tables

**Table 1:** Hydrogen-bond geometry (Å, °)

*D*—H⋯*A*	*D*—H	H⋯*A*	*D*⋯*A*	*D*—H⋯*A*
N1—H1*N*1⋯O1^i^	0.93 (3)	1.78 (3)	2.697 (2)	170 (2)
N2—H1*N*2⋯O2^ii^	1.01 (3)	1.89 (3)	2.889 (3)	167 (3)
N2—H2*N*2⋯O1^i^	0.83 (3)	2.48 (2)	3.144 (3)	138 (2)
N3—H1*N*3⋯O2	0.86 (3)	2.56 (3)	3.090 (2)	120 (2)
N3—H1*N*3⋯O3	0.86 (3)	2.18 (3)	3.005 (3)	161 (3)
N3—H2*N*3⋯O2^i^	0.89 (3)	2.02 (3)	2.892 (2)	168 (2)
N4—H1*N*4⋯O3	0.96 (3)	1.69 (3)	2.641 (2)	169 (3)
N5—H1*N*5⋯O3	0.88 (3)	2.53 (3)	3.208 (3)	135 (2)
N5—H2*N*5⋯O1^iii^	0.91 (3)	2.00 (3)	2.886 (3)	166 (2)
N6—H1*N*6⋯O4	0.92 (3)	1.96 (3)	2.866 (2)	169 (2)
N6—H2*N*6⋯O4^iv^	0.87 (3)	2.02 (3)	2.824 (2)	155 (3)
N8—H8*A*⋯O4^ii^	0.86	2.35	3.196 (4)	170
C15—H15*A*⋯O3^iii^	0.93	2.58	3.422 (3)	151
C10—H10*A*⋯*Cg*1^v^	0.93	2.48	3.379 (3)	163

Symmetry codes: (i) 
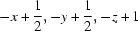
; (ii) 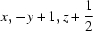; (iii) 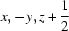; (iv) 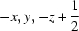; (v) 
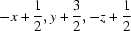
. *Cg*1 is the centroid of the C1–C6 ring.

**Fig. 1.**
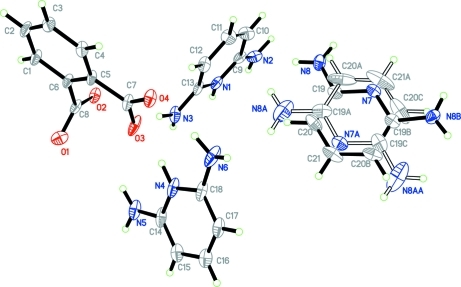
The molecular structure of the title compound, showing 30% probability displacement ellipsoids and the atom-numbering scheme. Open bonds indicate the minor disordered component.

**Fig. 2.**
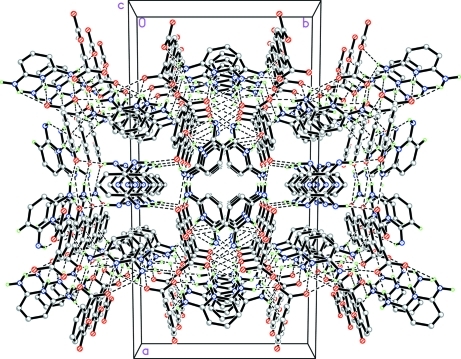
The crystal packing of the title compound, viewed along the c axis, showing the three-dimensional network. Only major components are shown. H atoms not involved in intermolecular interactions (dashed lines) have been omitted for clarity.

## supplementary crystallographic information

### Comment

2,6-Diaminopyridinium and diaminopyridine in general have an important role in the preparation of aromatic azo dyes, the subject of many polarographic investigations (Abu Zuhri & Cox, 1989). It has amino-imino tautomerization property (Inuzuka & Fujimoto, 1990) and it can be used to prepare polymer complexes with lead(II), cadmium(II) and zinc(II) (El-Mossalamy, 2001). Phthalic acid is an aromatic dicarboxylic acid and can be used in its anhydride form to produce other chemicals such as dyes, perfumes, phthalates and many others. It can be prepared from the catalytic oxidation of naphthalene in a new production method (Brike *et al.*, 2002). Some of its derivatives have anti-tumor promoting action (Yamamoto *et al.*, 1990). The crystal structure of this molecule can be helpful in future experimental and theoretical studies.

The asymmetric unit of the title salt (Fig. 1) contains two 2,6-diaminopyridine cations, one phthalate anion and a half 2,6-diaminopyridine molecule. The 2,6-diaminopyridine molecule has a twofold rotation symmetry. Atom N7 and C21 of the major component and N7A and C21A of the minor component lie across the crystallographic twofold rotation symmetry [suffix A corresponds to the symmetry code = -*x*, *y*, 1/2 - *z*]. Two protons are transferred from the carboxyl groups of the phthalic acid to atoms N1 and N4 of the 2,6-diaminopyridine moieties resulting in the formation of organic salts. The 2,6-diamionopyridine molecule is disordered over two positions with a site-occupancy ratio of 0.534 (3):0.466 (3). Both the N1/C9–C13 and N4/C14–C18 pyridine rings are essentially planar, with maximum deviations of 0.011 (2) Å at C12 and 0.006 (2) Å at C14, respectively. These two rings are inclined to one another with a dihedral angle of 79.86 (10)°. In the phthalate anion, the torsion angles of C1–C6–C8–O2 and C4–C5–C7–O3 are 88.3 (2) and -178.9 (2)°, respectively. The bond lengths (Allen *et al.*, 1987) and angles are within normal ranges and are comparable to a related structure (Büyükgüngör & Odabąsoǧlu, 2006).

This crystal structure is mainly stabilized by a network of N—H···O and C—H···O hydrogen bonds. The N atoms of the diaminopyridine cations provide the most extensive part as donors. In the crystal structure (Fig. 2), intermolecular N1—H1N1···O1, N2—H1N2···O2, N2—H2N2···O1, N3—H1N3···O2, N3—H1N3···O3, N3—H2N3···O2, N4—H1N4···O3, N5—H1N5···O3, N5—H2N5···O1, N6—H1N6···O4, N6—H2N6···O4, N8—H8A···O4 and C15—H15A···O3 hydrogen bonds (Table 1) link the structure into a three-dimensional network. This structure is further stabilized by weak intermolecular C—H···π (Table 1) interactions involving the C1–C6 (Centroid *Cg*1) ring.

### Experimental

In order to prepare the title crystal, phthalic acid (0.01 mol, 1.75 g) was dissolved in 25 ml of THF in a round bottom flask. In a separating funnel, 2,6-diaminopyridine (0.03 mol, 3.75 g) was dissolved in 20 ml of THF. 2,6-Diaminopyridine solution was added in drops to the flask of phthalic acid solution with stirring. The reaction mixture was left stirring for 3 h at room temperature. Colourless crystals were separated, washed with THF and dried at 80°C.

### Refinement

Hydrogen atoms attached to nitrogen atoms (excepting for H8A, H8B, H8AA and H8AB) were located in a difference Fourier map. H8A, H8B, H8AA, H8AB and all the hydrogen atoms attached to carbon atoms were positioned geometrically [N–H = 0.86 Å, C–H = 0.93 Å] and were refined using a riding model, with *U*_iso_(H) = 1.2 *U*_eq_(N, C). Rigid, similarity and simulation restraints were applied to the disordered diaminopyridine ring.

### Crystal data

**Table:**

4C_5_H_8_N_3_^+^·2C_8_H_4_O_4_^2^^−^·C_5_H_7_N_3_	*F*(000) = 1848
*M**_r_* = 877.94	*D*_x_ = 1.349 Mg m^−^^3^
Monoclinic, *C*2/*c*	Mo *K*α radiation, λ = 0.71073 Å
Hall symbol: -C 2yc	Cell parameters from 8039 reflections
*a* = 29.7011 (6) Å	θ = 2.5–29.2°
*b* = 15.2183 (3) Å	µ = 0.10 mm^−^^1^
*c* = 9.7666 (2) Å	*T* = 100 K
β = 101.670 (1)°	Plate, colourless
*V* = 4323.25 (15) Å^3^	0.65 × 0.19 × 0.08 mm
*Z* = 4	

### Data collection

**Table:**

Bruker SMART APEXII CCD area-detector diffractometer	6403 independent reflections
Radiation source: fine-focus sealed tube	3722 reflections with *I* > 2σ(*I*)
graphite	*R*_int_ = 0.037
φ and ω scans	θ_max_ = 30.3°, θ_min_ = 2.5°
Absorption correction: multi-scan (*SADABS*; Bruker, 2005)	*h* = −41→33
*T*_min_ = 0.939, *T*_max_ = 0.992	*k* = −18→21
28159 measured reflections	*l* = −13→13

### Refinement

**Table:**

Refinement on *F*^2^	Primary atom site location: structure-invariant direct methods
Least-squares matrix: full	Secondary atom site location: difference Fourier map
*R*[*F*^2^ > 2σ(*F*^2^)] = 0.064	Hydrogen site location: inferred from neighbouring sites
*wR*(*F*^2^) = 0.153	H atoms treated by a mixture of independent and constrained refinement
*S* = 1.05	*w* = 1/[σ^2^(*F*_o_^2^) + (0.0492*P*)^2^ + 4.6062*P*] where *P* = (*F*_o_^2^ + 2*F*_c_^2^)/3
6403 reflections	(Δ/σ)_max_ = 0.001
368 parameters	Δρ_max_ = 0.41 e Å^−^^3^
138 restraints	Δρ_min_ = −0.29 e Å^−^^3^

### Special details

**Table:**

Experimental. The crystal was placed in the cold stream of an Oxford Cyrosystems Cobra open-flow nitrogen cryostat (Cosier & Glazer, 1986) operating at 100.0 (1) K.
Geometry. All e.s.d.'s (except the e.s.d. in the dihedral angle between two l.s. planes) are estimated using the full covariance matrix. The cell e.s.d.'s are taken into account individually in the estimation of e.s.d.'s in distances, angles and torsion angles; correlations between e.s.d.'s in cell parameters are only used when they are defined by crystal symmetry. An approximate (isotropic) treatment of cell e.s.d.'s is used for estimating e.s.d.'s involving l.s. planes.
Refinement. Refinement of *F*^2^ against ALL reflections. The weighted *R*-factor *wR* and goodness of fit *S* are based on *F*^2^, conventional *R*-factors *R* are based on *F*, with *F* set to zero for negative *F*^2^. The threshold expression of *F*^2^ > σ(*F*^2^) is used only for calculating *R*-factors(gt) *etc*. and is not relevant to the choice of reflections for refinement. *R*-factors based on *F*^2^ are statistically about twice as large as those based on *F*, and *R*- factors based on ALL data will be even larger.

### Fractional atomic coordinates and isotropic or equivalent isotropic displacement parameters (Å^2^)

**Table:**

	*x*	*y*	*z*	*U*_iso_*/*U*_eq_	Occ. (<1)
O1	0.21274 (4)	0.08852 (11)	0.21602 (15)	0.0440 (4)	
O2	0.22943 (4)	0.23084 (10)	0.25406 (14)	0.0387 (4)	
O3	0.13432 (4)	0.19360 (12)	0.30278 (14)	0.0515 (5)	
O4	0.06579 (4)	0.23383 (10)	0.18270 (14)	0.0388 (4)	
C1	0.18725 (7)	0.19537 (15)	−0.0669 (2)	0.0423 (5)	
H1A	0.2177	0.1810	−0.0672	0.051*	
C2	0.15801 (8)	0.21910 (16)	−0.1906 (2)	0.0456 (6)	
H2A	0.1689	0.2210	−0.2734	0.055*	
C3	0.11279 (7)	0.23982 (14)	−0.1911 (2)	0.0405 (5)	
H3A	0.0932	0.2558	−0.2742	0.049*	
C4	0.09662 (6)	0.23687 (14)	−0.0685 (2)	0.0348 (5)	
H4A	0.0660	0.2506	−0.0698	0.042*	
C5	0.12550 (6)	0.21362 (13)	0.05745 (18)	0.0300 (4)	
C6	0.17151 (6)	0.19283 (14)	0.05818 (18)	0.0325 (5)	
C7	0.10699 (6)	0.21365 (14)	0.18992 (19)	0.0342 (5)	
C8	0.20650 (6)	0.16996 (16)	0.18790 (19)	0.0347 (5)	
N3	0.19685 (7)	0.30697 (15)	0.5108 (2)	0.0480 (5)	
N1	0.20894 (5)	0.44706 (13)	0.59887 (17)	0.0355 (4)	
N2	0.22822 (6)	0.58214 (16)	0.7028 (2)	0.0431 (5)	
C9	0.20034 (6)	0.53472 (15)	0.6039 (2)	0.0369 (5)	
C10	0.16395 (7)	0.56915 (17)	0.5066 (2)	0.0475 (6)	
H10A	0.1572	0.6289	0.5059	0.057*	
C11	0.13800 (7)	0.51321 (17)	0.4111 (2)	0.0497 (6)	
H11A	0.1137	0.5363	0.3458	0.060*	
C12	0.14655 (7)	0.42488 (17)	0.4086 (2)	0.0447 (6)	
H12A	0.1280	0.3884	0.3444	0.054*	
C13	0.18370 (6)	0.39106 (16)	0.50433 (19)	0.0384 (5)	
N4	0.09366 (6)	0.11944 (16)	0.49015 (18)	0.0464 (5)	
N5	0.16327 (6)	0.04646 (18)	0.5394 (2)	0.0502 (6)	
N6	0.02842 (7)	0.20371 (18)	0.4271 (2)	0.0634 (7)	
C14	0.12044 (6)	0.05699 (17)	0.5647 (2)	0.0448 (6)	
C15	0.10308 (7)	0.00880 (16)	0.6624 (2)	0.0455 (6)	
H15A	0.1209	−0.0338	0.7163	0.055*	
C16	0.05859 (7)	0.02544 (17)	0.6783 (2)	0.0467 (6)	
H16A	0.0466	−0.0071	0.7433	0.056*	
C17	0.03147 (7)	0.08875 (16)	0.6008 (2)	0.0453 (6)	
H17A	0.0016	0.0985	0.6125	0.054*	
C18	0.04969 (6)	0.13754 (17)	0.5051 (2)	0.0456 (6)	
N7	0.0000	0.5548 (4)	0.7500	0.0334 (16)	0.534 (5)
N8	0.05683 (11)	0.5598 (2)	0.6223 (3)	0.0411 (11)	0.534 (5)
H8A	0.0563	0.6162	0.6294	0.049*	0.534 (5)
H8B	0.0756	0.5352	0.5776	0.049*	0.534 (5)
C19	0.0278 (2)	0.5094 (5)	0.6826 (7)	0.0349 (15)	0.534 (5)
C20	0.0267 (4)	0.4174 (5)	0.6760 (14)	0.049 (2)	0.534 (5)
H20A	0.0440	0.3872	0.6219	0.059*	0.534 (5)
C21	0.0000	0.3734 (7)	0.7500	0.051 (3)	0.534 (5)
H21A	0.0000	0.3123	0.7500	0.061*	0.534 (5)
N7A	0.0000	0.4041 (6)	0.7500	0.044 (2)	0.466 (5)
N8A	0.04699 (18)	0.4030 (5)	0.5906 (6)	0.102 (2)	0.466 (5)
H8AA	0.0457	0.3466	0.5913	0.122*	0.466 (5)
H8AB	0.0629	0.4293	0.5384	0.122*	0.466 (5)
C19A	0.0240 (4)	0.4504 (6)	0.6714 (13)	0.050 (2)	0.466 (5)
C20A	0.0241 (4)	0.5423 (6)	0.6703 (13)	0.081 (3)	0.466 (5)
H20B	0.0408	0.5729	0.6148	0.097*	0.466 (5)
C21A	0.0000	0.5853 (10)	0.7500	0.093 (5)	0.466 (5)
H21B	0.0000	0.6464	0.7500	0.111*	0.466 (5)
H1N1	0.2349 (8)	0.4278 (15)	0.662 (3)	0.055 (7)*	
H1N2	0.2239 (10)	0.648 (2)	0.711 (3)	0.084 (10)*	
H2N2	0.2508 (8)	0.5584 (15)	0.754 (3)	0.046 (7)*	
H1N3	0.1847 (9)	0.2727 (17)	0.443 (3)	0.057 (8)*	
H2N3	0.2216 (8)	0.2915 (15)	0.574 (3)	0.046 (6)*	
H1N4	0.1058 (10)	0.1524 (19)	0.422 (3)	0.078 (9)*	
H1N5	0.1696 (8)	0.0712 (16)	0.464 (3)	0.053 (8)*	
H2N5	0.1805 (10)	0.0010 (18)	0.582 (3)	0.069 (9)*	
H1N6	0.0413 (9)	0.2208 (17)	0.353 (3)	0.062 (7)*	
H2N6	−0.0011 (9)	0.2076 (16)	0.419 (3)	0.059 (7)*	

### Atomic displacement parameters (Å^2^)

**Table:**

	*U*^11^	*U*^22^	*U*^33^	*U*^12^	*U*^13^	*U*^23^
O1	0.0227 (7)	0.0637 (11)	0.0404 (9)	0.0031 (7)	−0.0057 (6)	0.0151 (8)
O2	0.0254 (6)	0.0651 (10)	0.0233 (7)	0.0144 (7)	−0.0008 (5)	−0.0041 (7)
O3	0.0221 (6)	0.1140 (14)	0.0178 (7)	0.0141 (8)	0.0020 (5)	−0.0077 (8)
O4	0.0169 (6)	0.0635 (10)	0.0354 (8)	0.0058 (6)	0.0042 (5)	−0.0088 (7)
C1	0.0341 (10)	0.0693 (16)	0.0254 (10)	0.0190 (10)	0.0105 (8)	0.0004 (10)
C2	0.0552 (13)	0.0645 (16)	0.0183 (10)	0.0146 (11)	0.0105 (9)	−0.0021 (10)
C3	0.0463 (12)	0.0493 (13)	0.0200 (10)	0.0113 (10)	−0.0078 (8)	−0.0049 (9)
C4	0.0244 (9)	0.0490 (13)	0.0261 (10)	0.0089 (8)	−0.0067 (7)	−0.0089 (9)
C5	0.0206 (8)	0.0492 (12)	0.0185 (9)	0.0091 (8)	−0.0002 (7)	−0.0075 (8)
C6	0.0250 (9)	0.0527 (13)	0.0192 (9)	0.0120 (8)	0.0034 (7)	−0.0010 (8)
C7	0.0198 (8)	0.0584 (14)	0.0231 (9)	0.0049 (8)	0.0014 (7)	−0.0111 (9)
C8	0.0152 (8)	0.0696 (16)	0.0195 (9)	0.0125 (9)	0.0041 (7)	0.0045 (10)
N3	0.0394 (10)	0.0767 (16)	0.0229 (9)	0.0302 (10)	−0.0055 (8)	−0.0076 (10)
N1	0.0186 (7)	0.0658 (13)	0.0223 (8)	0.0113 (8)	0.0044 (6)	0.0089 (8)
N2	0.0203 (8)	0.0636 (14)	0.0411 (11)	−0.0034 (8)	−0.0041 (8)	0.0173 (10)
C9	0.0190 (8)	0.0614 (15)	0.0307 (10)	0.0044 (9)	0.0062 (8)	0.0125 (10)
C10	0.0280 (10)	0.0582 (15)	0.0502 (14)	0.0066 (10)	−0.0063 (10)	0.0122 (12)
C11	0.0271 (10)	0.0734 (18)	0.0424 (13)	0.0163 (11)	−0.0076 (9)	0.0089 (12)
C12	0.0295 (10)	0.0721 (17)	0.0286 (11)	0.0187 (10)	−0.0030 (8)	−0.0025 (11)
C13	0.0264 (9)	0.0711 (16)	0.0184 (9)	0.0164 (10)	0.0063 (8)	0.0009 (10)
N4	0.0241 (8)	0.0920 (16)	0.0210 (8)	0.0174 (9)	−0.0002 (7)	−0.0105 (9)
N5	0.0266 (9)	0.0893 (17)	0.0310 (10)	0.0215 (10)	−0.0029 (8)	−0.0127 (11)
N6	0.0260 (9)	0.133 (2)	0.0329 (11)	0.0320 (11)	0.0098 (8)	0.0162 (12)
C14	0.0260 (9)	0.0796 (17)	0.0239 (10)	0.0151 (10)	−0.0068 (8)	−0.0214 (11)
C15	0.0350 (11)	0.0620 (16)	0.0349 (12)	0.0106 (10)	−0.0042 (9)	−0.0145 (11)
C16	0.0337 (11)	0.0639 (16)	0.0404 (12)	0.0016 (10)	0.0023 (9)	−0.0155 (11)
C17	0.0235 (9)	0.0747 (17)	0.0361 (12)	0.0056 (10)	0.0023 (9)	−0.0184 (11)
C18	0.0217 (9)	0.0897 (18)	0.0231 (10)	0.0156 (10)	−0.0009 (8)	−0.0139 (11)
N7	0.025 (3)	0.032 (4)	0.037 (3)	0.000	−0.006 (2)	0.000
N8	0.0274 (17)	0.056 (2)	0.040 (2)	0.0022 (15)	0.0074 (14)	−0.0074 (16)
C19	0.021 (2)	0.039 (4)	0.037 (3)	0.002 (3)	−0.0130 (19)	0.002 (3)
C20	0.041 (3)	0.032 (4)	0.066 (5)	0.005 (3)	−0.012 (3)	−0.012 (4)
C21	0.032 (4)	0.031 (6)	0.073 (6)	0.000	−0.030 (3)	0.000
N7A	0.044 (4)	0.035 (6)	0.047 (4)	0.000	−0.002 (3)	0.000
N8A	0.060 (3)	0.188 (7)	0.061 (4)	0.063 (4)	0.020 (3)	0.054 (4)
C19A	0.036 (4)	0.055 (6)	0.050 (4)	−0.008 (6)	−0.016 (3)	0.014 (6)
C20A	0.062 (6)	0.063 (6)	0.091 (6)	−0.028 (5)	−0.048 (4)	0.048 (5)
C21A	0.083 (8)	0.038 (8)	0.120 (10)	0.000	−0.070 (6)	0.000

### Geometric parameters (Å, °)

**Table:**

O1—C8	1.275 (3)	N4—H1N4	0.96 (3)
O2—C8	1.250 (3)	N5—C14	1.353 (3)
O3—C7	1.267 (2)	N5—H1N5	0.88 (3)
O4—C7	1.250 (2)	N5—H2N5	0.91 (3)
C1—C2	1.386 (3)	N6—C18	1.341 (3)
C1—C6	1.394 (2)	N6—H1N6	0.92 (3)
C1—H1A	0.9300	N6—H2N6	0.87 (3)
C2—C3	1.379 (3)	C14—C15	1.384 (3)
C2—H2A	0.9300	C15—C16	1.385 (3)
C3—C4	1.378 (3)	C15—H15A	0.9300
C3—H3A	0.9300	C16—C17	1.380 (3)
C4—C5	1.396 (3)	C16—H16A	0.9300
C4—H4A	0.9300	C17—C18	1.387 (3)
C5—C6	1.401 (2)	C17—H17A	0.9300
C5—C7	1.505 (2)	N7—C19^i^	1.346 (6)
C6—C8	1.508 (3)	N7—C19	1.346 (6)
N3—C13	1.336 (3)	N8—C19	1.373 (7)
N3—H1N3	0.86 (3)	N8—H8A	0.8600
N3—H2N3	0.89 (2)	N8—H8B	0.8600
N1—C9	1.361 (3)	C19—C20	1.401 (7)
N1—C13	1.364 (3)	C20—C21	1.354 (10)
N1—H1N1	0.93 (3)	C20—H20A	0.9300
N2—C9	1.347 (3)	C21—C20^i^	1.354 (10)
N2—H1N2	1.01 (3)	C21—H21A	0.9300
N2—H2N2	0.83 (2)	N7A—C19A^i^	1.348 (10)
C9—C10	1.389 (3)	N7A—C19A	1.348 (10)
C10—C11	1.378 (3)	N8A—C19A	1.351 (10)
C10—H10A	0.9300	N8A—H8AA	0.8600
C11—C12	1.369 (3)	N8A—H8AB	0.8600
C11—H11A	0.9300	C19A—C20A	1.398 (10)
C12—C13	1.393 (3)	C20A—C21A	1.332 (12)
C12—H12A	0.9300	C20A—H20B	0.9300
N4—C14	1.353 (3)	C21A—C20A^i^	1.332 (12)
N4—C18	1.371 (2)	C21A—H21B	0.9300
			
C2—C1—C6	120.62 (18)	C14—N4—H1N4	118.5 (17)
C2—C1—H1A	119.7	C18—N4—H1N4	117.9 (17)
C6—C1—H1A	119.7	C14—N5—H1N5	118.0 (16)
C3—C2—C1	120.05 (18)	C14—N5—H2N5	118.2 (17)
C3—C2—H2A	120.0	H1N5—N5—H2N5	121 (2)
C1—C2—H2A	120.0	C18—N6—H1N6	115.7 (16)
C4—C3—C2	120.00 (18)	C18—N6—H2N6	117.2 (17)
C4—C3—H3A	120.0	H1N6—N6—H2N6	119 (2)
C2—C3—H3A	120.0	N5—C14—N4	117.2 (2)
C3—C4—C5	120.95 (17)	N5—C14—C15	123.9 (2)
C3—C4—H4A	119.5	N4—C14—C15	118.87 (18)
C5—C4—H4A	119.5	C14—C15—C16	118.5 (2)
C4—C5—C6	119.12 (16)	C14—C15—H15A	120.7
C4—C5—C7	119.45 (15)	C16—C15—H15A	120.7
C6—C5—C7	121.41 (16)	C17—C16—C15	122.0 (2)
C1—C6—C5	119.26 (17)	C17—C16—H16A	119.0
C1—C6—C8	116.51 (15)	C15—C16—H16A	119.0
C5—C6—C8	124.20 (15)	C16—C17—C18	118.67 (19)
O4—C7—O3	123.78 (17)	C16—C17—H17A	120.7
O4—C7—C5	118.39 (16)	C18—C17—H17A	120.7
O3—C7—C5	117.83 (15)	N6—C18—N4	116.1 (2)
O2—C8—O1	124.68 (17)	N6—C18—C17	125.55 (18)
O2—C8—C6	118.3 (2)	N4—C18—C17	118.3 (2)
O1—C8—C6	116.80 (19)	C19^i^—N7—C19	118.3 (7)
C13—N3—H1N3	118.0 (17)	C19—N8—H8A	120.0
C13—N3—H2N3	118.5 (15)	C19—N8—H8B	120.0
H1N3—N3—H2N3	122 (2)	H8A—N8—H8B	120.0
C9—N1—C13	123.73 (17)	N7—C19—N8	115.0 (5)
C9—N1—H1N1	114.9 (15)	N7—C19—C20	121.6 (7)
C13—N1—H1N1	121.2 (15)	N8—C19—C20	123.4 (7)
C9—N2—H1N2	121.1 (17)	C21—C20—C19	118.7 (9)
C9—N2—H2N2	120.2 (17)	C21—C20—H20A	120.6
H1N2—N2—H2N2	119 (2)	C19—C20—H20A	120.6
N2—C9—N1	117.26 (18)	C20^i^—C21—C20	120.7 (10)
N2—C9—C10	124.7 (2)	C20^i^—C21—H21A	119.7
N1—C9—C10	118.1 (2)	C20—C21—H21A	119.7
C11—C10—C9	118.8 (2)	C19A^i^—N7A—C19A	117.0 (10)
C11—C10—H10A	120.6	C19A—N8A—H8AA	120.0
C9—C10—H10A	120.6	C19A—N8A—H8AB	120.0
C12—C11—C10	122.6 (2)	H8AA—N8A—H8AB	120.0
C12—C11—H11A	118.7	N7A—C19A—N8A	116.2 (8)
C10—C11—H11A	118.7	N7A—C19A—C20A	121.9 (10)
C11—C12—C13	118.4 (2)	N8A—C19A—C20A	121.8 (10)
C11—C12—H12A	120.8	C21A—C20A—C19A	119.0 (12)
C13—C12—H12A	120.8	C21A—C20A—H20B	120.5
N3—C13—N1	116.81 (18)	C19A—C20A—H20B	120.5
N3—C13—C12	124.8 (2)	C20A—C21A—C20A^i^	121.1 (14)
N1—C13—C12	118.4 (2)	C20A—C21A—H21B	119.5
C14—N4—C18	123.6 (2)	C20A^i^—C21A—H21B	119.5
			
C6—C1—C2—C3	−0.5 (4)	C9—N1—C13—N3	178.83 (17)
C1—C2—C3—C4	−0.1 (4)	C9—N1—C13—C12	−1.0 (3)
C2—C3—C4—C5	0.4 (3)	C11—C12—C13—N3	−177.8 (2)
C3—C4—C5—C6	−0.2 (3)	C11—C12—C13—N1	2.0 (3)
C3—C4—C5—C7	178.2 (2)	C18—N4—C14—N5	−179.7 (2)
C2—C1—C6—C5	0.7 (3)	C18—N4—C14—C15	−0.6 (3)
C2—C1—C6—C8	−177.5 (2)	N5—C14—C15—C16	−179.8 (2)
C4—C5—C6—C1	−0.4 (3)	N4—C14—C15—C16	1.1 (3)
C7—C5—C6—C1	−178.8 (2)	C14—C15—C16—C17	−0.6 (3)
C4—C5—C6—C8	177.7 (2)	C15—C16—C17—C18	−0.6 (3)
C7—C5—C6—C8	−0.7 (3)	C14—N4—C18—N6	177.5 (2)
C4—C5—C7—O4	0.7 (3)	C14—N4—C18—C17	−0.6 (3)
C6—C5—C7—O4	179.11 (19)	C16—C17—C18—N6	−176.8 (2)
C4—C5—C7—O3	−178.9 (2)	C16—C17—C18—N4	1.2 (3)
C6—C5—C7—O3	−0.5 (3)	C19^i^—N7—C19—N8	−176.8 (7)
C1—C6—C8—O2	88.3 (2)	C19^i^—N7—C19—C20	3.0 (8)
C5—C6—C8—O2	−89.8 (2)	N7—C19—C20—C21	−6.0 (16)
C1—C6—C8—O1	−86.7 (2)	N8—C19—C20—C21	173.8 (7)
C5—C6—C8—O1	95.2 (2)	C19—C20—C21—C20^i^	2.9 (8)
C13—N1—C9—N2	−179.82 (16)	C19A^i^—N7A—C19A—N8A	−178.4 (12)
C13—N1—C9—C10	−0.4 (3)	C19A^i^—N7A—C19A—C20A	−0.2 (8)
N2—C9—C10—C11	−179.8 (2)	N7A—C19A—C20A—C21A	0.4 (17)
N1—C9—C10—C11	0.8 (3)	N8A—C19A—C20A—C21A	178.5 (9)
C9—C10—C11—C12	0.2 (3)	C19A—C20A—C21A—C20A^i^	−0.2 (8)
C10—C11—C12—C13	−1.6 (3)		

Symmetry codes: (i) −*x*, *y*, −*z*+3/2.

### Hydrogen-bond geometry (Å, °)

**Table:**

*D*—H···*A*	*D*—H	H···*A*	*D*···*A*	*D*—H···*A*
N1—H1N1···O1^ii^	0.93 (3)	1.78 (3)	2.697 (2)	170 (2)
N2—H1N2···O2^iii^	1.01 (3)	1.89 (3)	2.889 (3)	167 (3)
N2—H2N2···O1^ii^	0.83 (3)	2.48 (2)	3.144 (3)	138 (2)
N3—H1N3···O2	0.86 (3)	2.56 (3)	3.090 (2)	120 (2)
N3—H1N3···O3	0.86 (3)	2.18 (3)	3.005 (3)	161 (3)
N3—H2N3···O2^ii^	0.89 (3)	2.02 (3)	2.892 (2)	168 (2)
N4—H1N4···O3	0.96 (3)	1.69 (3)	2.641 (2)	169 (3)
N5—H1N5···O3	0.88 (3)	2.53 (3)	3.208 (3)	135 (2)
N5—H2N5···O1^iv^	0.91 (3)	2.00 (3)	2.886 (3)	166 (2)
N6—H1N6···O4	0.92 (3)	1.96 (3)	2.866 (2)	169 (2)
N6—H2N6···O4^v^	0.87 (3)	2.02 (3)	2.824 (2)	155 (3)
N8—H8A···O4^iii^	0.86	2.35	3.196 (4)	170
C15—H15A···O3^iv^	0.93	2.58	3.422 (3)	151
C10—H10A···Cg1^vi^	0.93	2.48	3.379 (3)	163

Symmetry codes: (ii) −*x*+1/2, −*y*+1/2, −*z*+1; (iii) *x*, −*y*+1, *z*+1/2; (iv) *x*, −*y*, *z*+1/2; (v) −*x*, *y*, −*z*+1/2; (vi) −*x*+1/2, *y*+3/2, −*z*+1/2.
